# The Response of Living Organisms to Low Radiation Environment and Its Implications in Radiation Protection

**DOI:** 10.3389/fpubh.2020.601711

**Published:** 2020-12-15

**Authors:** Mauro Belli, Luca Indovina

**Affiliations:** ^1^Independent Researcher, Rome, Italy; ^2^Dipartimento di Diagnostica per Immagini, Radioterapia Oncologica ed Ematologia, Fondazione Policlinico Universitario A. Gemelli IRCCS, Rome, Italy

**Keywords:** ionizing radiation, radiation protection, radiobiology, low dose effects of radiation, background radiation, epigenetics (MeSH), underground experiments

## Abstract

Life has evolved on Earth for about 4 billion years in the presence of the natural background of ionizing radiation. It is extremely likely that it contributed, and still contributes, to shaping present form of life. Today the natural background radiation is extremely small (few mSv/y), however it may be significant enough for living organisms to respond to it, perhaps keeping memory of this exposure. A better understanding of this response is relevant not only for improving our knowledge on life evolution, but also for assessing the robustness of the present radiation protection system at low doses, such as those typically encountered in everyday life. Given the large uncertainties in epidemiological data below 100 mSv, quantitative evaluation of these health risk is currently obtained with the aid of radiobiological models. These predict a health detriment, caused by radiation-induced genetic mutations, linearly related to the dose. However a number of studies challenged this paradigm by demonstrating the occurrence of non-linear responses at low doses, and of radioinduced epigenetic effects, i.e., heritable changes in genes expression not related to changes in DNA sequence. This review is focused on the role that epigenetic mechanisms, besides the genetic ones, can have in the responses to low dose and protracted exposures, particularly to natural background radiation. Many lines of evidence show that epigenetic modifications are involved in non-linear responses relevant to low doses, such as non-targeted effects and adaptive response, and that genetic and epigenetic effects share, in part, a common origin: the reactive oxygen species generated by ionizing radiation. Cell response to low doses of ionizing radiation appears more complex than that assumed for radiation protection purposes and that it is not always detrimental. Experiments conducted in underground laboratories with very low background radiation have even suggested positive effects of this background. Studying the changes occurring in various living organisms at reduced radiation background, besides giving information on the life evolution, have opened a new avenue to answer whether low doses are detrimental or beneficial, and to understand the relevance of radiobiological results to radiation protection.

## Introduction

Living organisms have evolved on Earth for about 4 billion years in the presence of the natural background of ionizing radiation even if it was not always the same as today. Without it, life on Earth could not have existed or would not exist in the present form. Not only the Earth's crust contains radionuclides, but also the Earth is continuously bombarded by high-energy particles originating in outer space and by the Sun (cosmic radiation).

In the late 20s, it was suggested that variations in cosmic radiation, in addition to possible contribution to organic evolution, could have affected the evolution of organic compounds and, eventually, of life on earth (1). It should be considered that probably in the past the cosmic rays on Earth have experienced many fluctuations due to explosions of supernovae in the nearby interstellar space and to variations in solar wind.

Today life is shielded against cosmic particles by the Earth's magnetic field and by the atmosphere layer but some radiation reaches the biosphere as a consequence of the primary particle interactions that generate secondary particles in the atmosphere. Understanding its role is important for improving our knowledge about life evolution on Earth and about the health effects of low dose ionizing radiation exposure, a hot topic in radiation protection. In this review the role of background radiation is considered in the perspective of the low dose issue in radiation protection.

## The Natural Background of Ionizing Radiation

Exposure of organisms to ionizing radiation from natural background is an unavoidable feature of life on Earth. The background radiation is intended as the radiation that is already in a location, when no source is deliberately introduced (2). The resulting doses to human beings have been evaluated for many years by international bodies, notably by the United Nations Scientific Committee on the Effects of Atomic Radiation (UNSCEAR), on the report of which the data here presented are based (3), with some integrations from the report issued by the U.S. National Academy of Sciences Biologic Effects of Ionizing Radiation, BEIR (4).

The main contribution to radiation background exposure of the public comes from natural sources of both low- and high-LET^1^, i.e., from cosmic radiation, external terrestrial radiation, inhalation and ingestion of radionuclides (Table 1), while the contribution due to man-made sources is today relatively small, mostly coming from the fall-out (now only about 0.005 mSv/year^2^ on average) of the nuclear weapons testing in the atmosphere occurred between 1945 and 1980.

**Table 1:** Average annual effective dose of public due to natural background exposure [based on data from UNSCEAR (3)] and BEIR (4).

**Source of exposure**	**World average** **(mSv)**	**Typical range** **(mSv)**	**Remark**
Cosmic radiation	0.39 (0.16%)	0.3–1.0	Depends on altitude, includes cosmogenic radionuclides
External terrestrial radiation	0.48 (0.20%)	0.3–1.0	Depends on soil and building material
Inhalation of air	1.26 (0.52%)	0.2–10	Mainly from radon, depends on indoor accumulation
Ingestion of food and water	0.29 (0.12%)	0.2–1.0	Depends on food and drinking water composition, includes K-40
Total (natural)	2.40	1.0–13	Depends strongly on the geographical site

Cosmic radiation originates by bombardment of the Earth by high-energy particles arising from outer space (galactic radiation) and by solar radiation (mainly protons, especially during solar flares). These particles interact with the nuclei of the atmosphere to produce a cascade of interactions and secondary reaction products that constitute the cosmic radiation on the Earth surface. These interactions also produce a number of radioactive nuclei known as cosmogenic radionuclides, such as C-14, that eventually reach the Earth's surface and can be incorporated into living organisms.

Besides the shielding provided by the earth's magnetic field, life is shielded against cosmic radiation by an air layer which is comparable at sea level to a 10 m thick water layer (3). Therefore, fluence and energy of these particles strongly depend on both latitude (the number of particles penetrating the atmosphere is higher close to the earth's poles) and altitude. At ground level, the cosmic ray field is largely from muons, neutrons, and electrons, with muons constituting the dominant component. In other words, it is made of various radiation qualities: roughly speaking, neutrons are high-LET particles, while muons, and other directly ionizing charged particles and photons, are low-LET radiation. Studying the effects of cosmic radiation on human organisms, even outside the protection offered by the earth's atmosphere and magnetic field, has presently a great importance for assessing the radiation risks during human space travel (6).

The main contribution to external terrestrial exposure comes from gamma-emitting radionuclides present in trace amounts in the soil, whose amount varies geographically.

Internal exposures arise from the intake of terrestrial radionuclides by inhalation and ingestion. Inhalation of radon gas and its decay products constitutes the majority of human exposure to background ionizing radiation. It results in lung irradiation by high-LET alpha particles, but also low-LET radiation is emitted. There are large differences in indoor radon concentration depending on the site, building characteristics, etc. Exposures from ingestion are mainly due to K-40 and to the U-238 and Th-232 series radionuclides present in food and drinking water and in the human body itself.

Today the annual dose due to external exposure from natural background on average approaches 1 mSv/y, with cosmic contributions slightly less than the terrestrial one. Contribution from inhalation is higher than external exposure (Table 1). It is worth noting here that: (i) the average annual dose from natural background of 2.4 mSv, corresponds to a low dose rate of ≈ 0.27 μSv/h; (ii) several areas of the world, such as Guarapan in Brazil, Ramsar in Iran, Yangjiang in China and Kerala in India, are found to have levels of natural background radiation that are in excess of those considered to be “normal background” (3)so that they are defined as High Natural Background Radiation (HNBR) areas (7); (iii) the evaluation of inhalation and ingestion exposures are strictly related to human beings and may not hold for different organisms, certainly not for the cultured cells often used in radiobiology experiments.

## The Low Dose Issue in Radiation Protection

### Epidemiological Approaches to Health Risks

Despite the fact that the natural radiation background is extremely small, nevertheless it may be significant enough for living organisms to sense it and respond to it, keeping memory of this exposure. Our knowledge about the response of living organisms to low and protracted doses of ionizing radiation is mainly related to radiation protection needs, where the focus is on detrimental effects. However, the response of living organisms to these levels of exposures is still a matter of debate.

Our scientific community has become aware of ionizing radiation just a little over a century ago, after the discovery of X-rays (1895) and of natural radioactivity (1896). Compared to other disciplines, radiation science is a quite recent field. Even if harmful effects were reported soon after the X-rays discovery, scientists were slow to understand them, probably because they were overshadowed by the enthusiastic attempts to treat with them nearly any kind of illness or discomfort.

In 1926, the American geneticist Muller discovered that, by exposing the fruit fly Drosophila melanogaster to high levels of radiation (such as X- or gamma-rays), the mutation rate in their offspring can be increased by as much as 150 times (8, 9). For this discovery he was awarded the 1946 Nobel Prize in medicine and physiology. His work convinced him that the vast majority of mutations were deleterious and consequently that exposure to radiation should be strictly controlled. However, the primary focus for radiation protection remained for long time the acute/deterministic radiation syndromes. It was only some years after the atomic bombing of Hiroshima and Nagasaki that increased radiation risks have been documented for malignant diseases among survivors, in particular leukemia (10). Since these health effects appeared as stochastic events, ICRP updated their recommendations, until then intended to keep exposures below the relevant thresholds for induction of deterministic effects, by introducing the concept of reducing exposure to the lowest possible level, a terminology today changed in “as low as reasonably achievable” (ALARA) level and included in the optimization principle.

Epidemiological studies consistently show that the main health risk at moderate and low doses (i.e., doses not causing acute/deterministic effects) is induction of solid tumors and leukemias and even today the most important long-term evaluation of populations exposed to radiation remains the epidemiological study of the Hiroshima and Nagasaki survivors of the atomic bombing (Life Span Study, LSS) with a cohort of about 120,000 subjects followed since 1950 (11). LSS also showed increased radiation risks for malignant diseases among survivors exposed *in utero*, and possible risks for some non-cancer diseases. Evidence for health effects of moderate/high-dose radiation (≥100 mSv), mostly consisting in linear dose-response relationship for cancer development, are conclusive, but the effects at lower dose levels are still uncertain (11).

**Figure 1:**
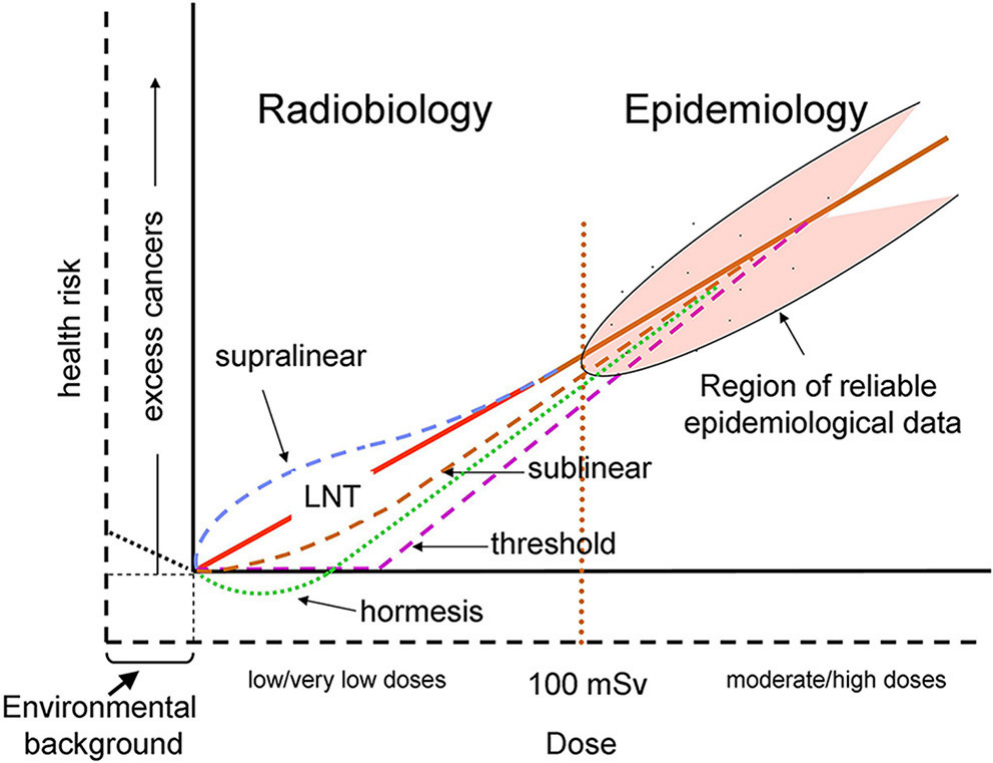
Schematic representation of possible dose–response relationships for radiation-induced cancer risk. The doses are effective doses in addition to the natural background exposure. The solid axes correspond to the current representation where doses are those above the natural background and the cancer risk is the corresponding excess risk (12), while the dashed axes also take into account the natural background. The vertical red dotted line at 100 mSv represents a rough separation between the regions of reliable (> ~100 mSv) and unreliable (< ~100 mSv) epidemiological data. The solid red line represents the conventional linear no-threshold (LNT) relationship. Hormetic response, represented by the dotted green line, corresponds to the hypothetical responses driven by doses above background, according to the usual representation. The dotted black line below background represents a hypothetical protective response driven by the background radiation. The other dashed colored lines represent various proposed dose–response relationships, that could take into account various non-linear responses, such as BE (often considered to induce a supralinear response), and AR (sublinear).

Indeed, for two perfectly matched populations of 100,000 people, the minimum detectable excess relative risk (ERR) is calculated to be around 0.05 for all cancers for whole population (12). Using the ERR for cancer induction derived from LSS of about 0.50 Gy-1 (13) the lowest dose with significant risk results to be about 100 mGy for this epidemiological study. Also, some recent epidemiological investigation have suggested possible cancer induction below this dose, but analytical limitations and other difficulties generated several controversies about their ability to provide firm epidemiological evidence of excess cancers at low doses (14). Given the limited statistical power and the large uncertainties in epidemiological data below 100 mGy, different plausible dose–response relationships could be considered for the risk of cancer from exposures at low and moderate doses (12) (Figure 1).

### What Is a “Low Dose” of Ionizing Radiation?

The term “low dose” has different definitions depending on the contexts. In the field of microdosimetry it is an absorbed dose such that a single cell or nucleus is very unlikely to be traversed by more than 1 track so that the number of affected nuclei or cells is proportional to the absorbed dose. However, the definition of “unlikely” is subjective so that, according to a more precise and conservative definition used in some radiobiological context, it corresponds to a mean number of 0.2 tracks per nucleus or cell (15), meaning that <2% of the cells will be subject to traversals by more than one radiation track. This would correspond to a dose of only 0.2 mGy of low-LET radiation (16). Using an epidemiology-based definition a low dose is a dose below which it is not possible to detect adverse health effects (17). For radiation protection purposes the International Commission for Radiation Protection, ICRP (18) defines low doses as those of 100 mSv or less for low-LET radiation, a value also consistent with that used by UNSCEAR (3) and by the U.S. National Academy of Sciences Biologic Effects of Ionizing Radiation (4). In practice, the doses typically encountered in the workplace, in the environment and in diagnostic medicine fall in the low dose range; also irradiation of normal tissues in radiotherapy may be included in this type of exposure. Moreover, in view of protracted exposures, a low dose rate has been defined as 0.1 mGy/min or less for low-LET radiation (19).

### Radiobiology Is Needed to Extrapolate Epidemiological Data to Low Doses

**Figure 2:**
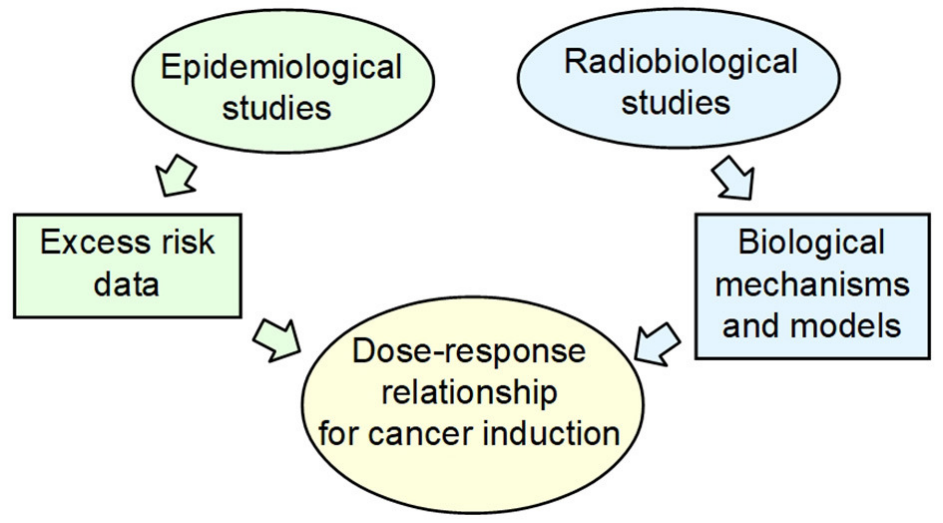
The current approach for health risk assessment at low and protracted dose, in particular for cancer induction, is a combination of epidemiological data and radiobiological mechanistic models.

Due to limitations of epidemiological data for excess of stochastic risk at low and protracted doses, radiobiological knowledge is recognized to provide a framework for the analysis of risks at low-dose and low-dose-rate exposures (20) and to establish causal relationships from correlations found by observational studies (21). The need of an integration between biological mechanisms and epidemiological approaches is invoked for assessing the shape of the dose–response relationship for cancer induction at low doses (19). In principle, radiobiology data and models can identify the relevant radiobiological mechanisms, thus providing a rationale for the extrapolation to low and protracted doses of the epidemiological data obtained at moderate and high doses (Figure 2).

Indeed, a wealth of radiobiological information has been obtained after just over a century of research about the response of living organisms to low dose of ionizing radiation. Since this research was stimulated by radiation protection needs, it is not surprising that the focus was on detrimental.

## The Radiobiological Bases of Health Risk Assessment

### The Conventional Paradigm of Radiobiology and the LNT Assumption

**Figure 3:**
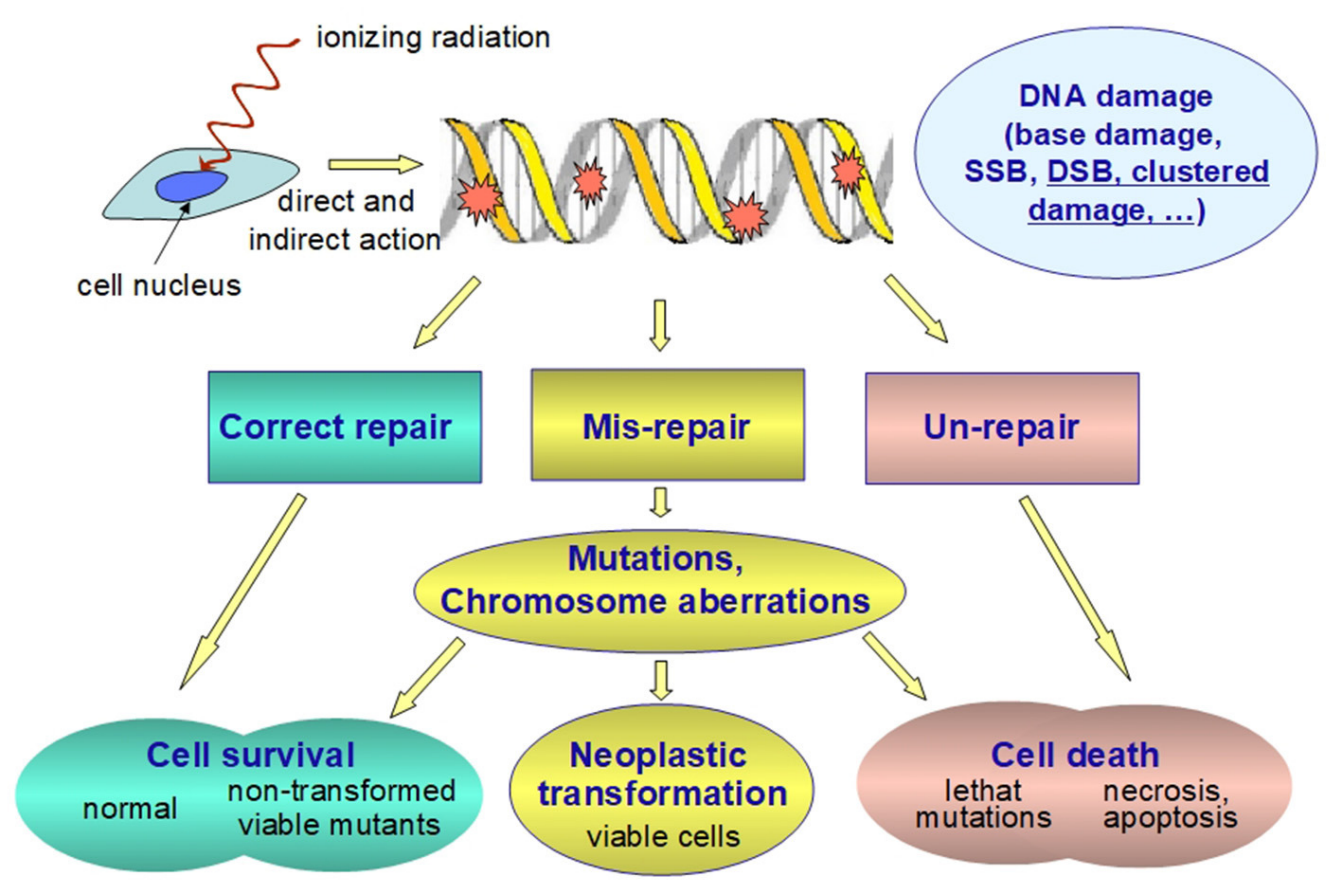
Schematic representation of the fate of radiation-induced DNA damage. Ionizing radiation can damage nuclear DNA by direct and indirect (i.e., through reactive chemical species formed as a result of the water radiolysis near the DNA) action. Clustered DNA lesions (defined as two or more lesions within one or two helical turns of DNA) are considered to be the most biologically relevant DNA damage since they are expected to be less readily repaired as compared to other damage. Unrepaired or misrepaired DNA lesions causes genetic mutations and/or chromosome aberrations, which are very likely detrimental. Even at low doses they are assumed to increase both the probability of developing cancer and the rates of hereditary diseases. Therefore, the health risk for stochastic effects arises not from the killed cells, but from the surviving cells bearing important genetic damage.

It is well-known that ionizing radiation, being defined as radiation capable to ionize biological matter (whose ionization threshold is ~12.4 eV), can break the chemical bonds of the cell components, the DNA being the most important cell target. Ionizing radiation can cause DNA lesion by direct deposition of energy in the DNA as well as by the indirect action through reactive chemical species (mostly free radicals) formed by radiolysis of water molecules near the DNA (22–24). Indirect DNA damage is the most frequent mechanisms for low-LET radiation while direct DNA damage is predominant for high-LET radiation (25, 26). The free radicals formed through the radiolysis of water molecules are converted, in aerobic conditions, to reactive oxygen species (ROS) that include free radicals as well as non-free radicals. Ionizing radiation can also generate reactive nitrogen species (RNS) through up-regulation of several enzymes, including inducible nitric oxide synthetase. The yield and spatial distribution of ROS and RNS is strongly modulated by radiation quality as a consequence of the specific track structure of each quality (27). ROS and RNS can attack the DNA resulting in several alterations, including DNA single strand and double strand breaks (SSB and DSB, respectively), base damage and destruction of sugars. The most biologically relevant radiation-induced DNA damage, both directly and indirectly, is the clustered DNA damage, defined as two or more DNA lesions formed within one or two helical turns of DNA (22, 24, 28, 29). Particularly relevant are complex DSB and non-DSB clusters (29–32). A complex DSB is a DSB with additional lesions within 10 bp. The proportion of complex DSB and the degree of complexity increase with increasing radiation LET (33). Indeed, ionizing radiation is uniquely very efficient at inducing clustered DNA lesions (34) compared to endogenous or metabolism-related cellular damage. At low doses, even the passage of a single particle can produce clustered DNA lesions (24, 29, 35). The degree of complexity is assumed to be an index of severity of the radiation-induced damage, since complex lesions are not repaired with high fidelity by the repair machinery of the cells. Unrepair or mis-repair may lead to genetic mutations in surviving cells (Figure 3). It is generally assumed that a vast majority of mutations are neutral or detrimental, as in many circumstances gene mutation is a process which acts contrary to natural selection and which burdens each population with a load of harmful genes (36). Cancer development is described as a multistep process originating from single cells that have sustained mutations through DNA damage and radiation is judged to act most commonly by inducing initiating mutations in proto-oncogenes or in tumor suppressor genes (12).

Based on the above considerations, the following overall picture has been developed, sometimes called “conventional paradigm of radiobiology” as described by Goodhead (37): (a) the DNA damage in directly exposed cells is the main event for biological effects; (b) the DNA damage occurs during, or very shortly after, irradiation of the nuclei in targeted cells; (c) the potential for biological consequences can be expressed within one or two cell generations; (d) at low doses the biological effect is in direct proportion to the energy deposited in nuclear DNA.

This paradigm implies that genetic mutations consequent to radiation-induced damage to the cellular DNA are the main event leading to deleterious biological effects and that they are linearly related to the absorbed dose. It provides therefore the rational basis for the assuming the LNT relationship between excess cancer risk and dose also at low doses. As far as this aspect is concerned, the internationally agreed system for radiation protection has developed from this paradigm, although with many simplifications and assumptions (18). LNT assumption makes radiation protection relatively easy and manageable (doses are additive, dose can be used as an index of risk).

In the last 15 years a lively debate developed on the health effects at low dose (14, 38–40), and consequently the LNT relationship was questioned using various arguments (41–45). In the following, the radiobiological bases relevant to this issue will be considered.

### Limitations of the Conventional Paradigm

The current approach in estimating health risks at low doses substantially uses a model centered on detrimental effects, arising from DNA damage and gene mutations, with a linear dose-response, similarly to that observed for the high-dose response. However, this model has been challenged since the end of 1980s from *in vitro* and *in vivo* studies that demonstrated the occurrence of biological responses inconsistent with it, in particular of non-linear dose-responses and non-genetic effects, such as those mentioned in the following.

### Biological Response Is Different Between High- and Low-Doses

Evidence is accumulated that living organisms, including humans, respond to low and protracted doses in a way that is not only quantitatively, but also qualitatively, different from that to high and acute dose. For example, low doses may be insufficient to induce an efficient DSB repair *in vitro* (46, 47), differences in gene expression profiles have been found in a human myeloid tumor cell line (48) and subsequently many other data were accumulated for a variety of biological systems (49–51), including human tissue models (52) and human tissue irradiated *in vivo* (53). These observations raise the question whether the dose-dependent biological response is reflected in non-linear relationship between health effects and dose. Moreover, in some circumstances human cellular responses to low doses of radiation were shown to induce lower levels of chromosomal damage than that occurring spontaneously at the basal level [(54) and refs therein (14) and refs therein], giving support to the assumption, based on studies with *in vitro* and animal models, that low-dose radiation has beneficial effects (55).

### Non-targeted and Adaptive Responses

A number of non-linear responses have been described in details in the last decades, such as the so-called non-targeted effects (NTE), i.e., effects that are not consequent to the DNA damage in the irradiated cells, and the adaptive response (AR). Remarkable NTE are the bystander effect (BE) (56, 57), the genomic instability (GI) (58–63), the low dose hypersensitivity (HRS) (64, 65), and delayed reproductive death (58) and induction of genes by radiation (66).

Particularly interesting and extensively studied NTE are the BE and the GI discovered between the end of the 1980s and the beginning of the 1990s (67–70) In general a BE describes the ability of cells affected by an agent to convey manifestations of damage to other cells not directly targeted by the agent or not necessarily susceptible to it *per se* (71). Thus, radiation induced BE is an effect manifesting in cells that were non-irradiated, neighbors of irradiated cells or that received factors secreted or shed by irradiated cells. Abscopal, or out-of-field, effects, defined in radiotherapy as radiation-induced effects observed outside the irradiated volume, are currently considered as a special type of BE (72–74).

GI is a delayed radiation effect, observed both *in vitro* and *in vivo* models, an all-embracing term to describe the increased rate of acquisition of alterations in the genome (62, 63). Radiation-induced genomic instability is characterized by an increased rate of genetic alterations ranging from simple DNA sequence changes to structural and numerical abnormalities at the chromosomal level in the progeny of irradiated cells multiple generations after the initial insult. It is measured, e.g., as enhanced death rate, chromosomal alterations, changes in ploidy, micronucleus formation, specific gene mutations.

AR can be regarded as a quite general phenomenon that can occur with various harmful agents. AR to radiation exposure is a transient phenomenon that has been observed in cells, tissues and organisms when a small conditioning radiation dose, called “priming dose,” reduces the biological effects of a subsequent (usually higher) radiation dose called “challenging dose” [see for a review (75)]. Early evidence of this effect was shown in human lymphocytes even earlier than NTE (76, 77).

In this review we will denote with BE and AR only those effects that are radiation-induced. NTE and AR have been observed in many *in-vitro and in-vivo* experiments, and also in *ex-vivo* experiments with blood samples from irradiated humans (63, 69). These phenomena have been seen using a variety of cell and tissue types, of biological end-points and of radiation qualities (69, 78–80), however they have not been universally observed (70, 81–83), an intriguing aspect that is not yet fully elucidated. All NTE can be described as the expression of inter- or intra-cellular signaling (84) and are deemed to be particularly relevant to cell response to low doses.

These studies, especially those on BE, support the notion that, in general, the cellular system responds as a whole and therefore, especially at low dose, the cell population must be seen as a single entity perturbed by radiation, and the response comes from the whole population in a coordinated way (85, 86). Interestingly, there is evidence that NTE and AR are inter-related (78, 87, 88) and likely related, at least in part, to non-DNA damage, so that epigenetic mechanisms may have a role in them (89, 90).

### Radiation-Induced Epigenetic Effects

Epigenetic changes are meiotically heritable and mitotically stable alterations in gene expression without alteration in DNA sequences and the “epigenome” can be intended as the complete set of the epigenetic trait of an organism. By the second half of the last century, it has been recognized that DNA by itself does not determine all characteristics of an organism, including the human one. Epigenetic regulation play an important role in various biological processes including embryonic development, genetic imprinting, and X-chromosome inactivation, therefore explaining how cells carrying identical DNA differentiate into different cell types, and how they maintain differentiated cellular states (91). Deregulation of these processes causes aberrant gene function and altered gene expression that play critical role in cancer initiation and development and, indeed, alterations of one or more of these processes are observed in various human cancers (92–96).

Environmental stimuli or stressors, including ionizing radiation, can trigger phenotype changes through epigenetic alterations [see the reviews in (97) and in (98)]. An interesting aspect is that some of them can be reversed after removal of the stressor (epigenetic plasticity) but some epigenetic modifications can persist (99, 100). A heritable change in gene expression induced by a previous stimulus is often called “epigenetic memory” (101).

Since the phenotype of a cell or individual is affected by which of its genes are transcribed, epigenetics is considered a bridge between genotype and phenotype. Genetic changes, such as mutations, are heritable, but not very affected by environmental influence (even if mutations can be induced by the environmental radiation, they are relatively rare events). At the other extreme there are the metabolic processes, susceptible to environmental changes, but not heritable. Epigenetic modifications, instead, are susceptible to environmental changes and are heritable at the same time (99).

The three key epigenetic processes are changes in DNA methylation, histones alteration leading to chromatin modification, and post-translational gene regulation by non-coding RNAs (ncRNAs).

DNA methylation represents the best characterized form of epigenetic modification. It is a covalent modification of the cytosine ring by addition of a methyl group at the 5′ position resulting in 5-metylcytosine (5 mC). In mammals, cytosine methylation is found almost exclusively in the context of CpG dinucleotides (97) and DNA methylation patterns are maintained or established by a family of enzymes, the DNA methyltransferases (DNMTs). DNA methylation establishes and maintains an inactive state of a gene by keeping the chromatin structure compact and inaccessible to the transcription machinery (102). However, it must be considered that 5 mC is inherently mutagenic because it can spontaneously undergo deamination, leading to C → T transitions. Typical alterations in DNA methylation consists in hypermethylation in specific genes, potentially leading to their transcriptional silencing, or in global hypomethylation, leading to genomic instability when transposable elements are de-methylated (103, 104). Although 5 mC was discovered in calf thymus DNA around the middle of the last century, it was only around 1980 that DNA methylation was demonstrated to be involved in gene regulation and cell differentiation (105). Not many years later, early findings indicated that exposure to ^60^Co ɤ-radiation causes a dose-dependent decreases in DNA methylation in several cultured cell lines (106). Since then, considerably amount of research carried out both *in vitro* and *in vivo* showed that exposure to ionizing radiation can change the DNA methylation pattern, both in terms of specific hypermethylation and/or global hypomethylation [see the reviews in (107, 108, 110)]. Such studies are relevant to a better understanding of radiation-induced cancer, as hypermethylation in the promoter region of a tumor-suppressor gene can cause its silencing, therefore contributing to the oncogenic process (103, 107–109, 111–113) while global DNA hypomethylation induces DNA hypomethylation, which plays critical roles in both cancer initiation and progression (114) being the first epigenetic alteration detected in several human cancers [see the reviews in (107) and (110)]. Indeed, hypermethylation of tumor-suppressor genes was observed in lung cancers of occupationally exposed workers at the Russian MAYAK plutonium plant [(115, 116) and references therein] and in an appreciable fraction of patients with renal cell carcinomas living in radiocontaminated areas after the Chernobyl accident (117). A link between radiation-induced global genomic hypomethylation and carcinogenesis was established in rats with radiation-induced mammary tumors [(118) see also the review in (108)].

In addition to DNA methylation changes, ionizing radiation was shown to induce a variety of histone modifications both *in vitro* and *in vivo*. Histones, as essential constituents of the chromatin in eukaryotic cells, were regarded for years as having a merely structural role. However, now they are recognized to control the organization of chromatin and hence transcriptional responses (119) In cultured cells and in mouse models exposure to ionizing radiation is shown to induce post-translational modification on histones, such as acetylation, methylation, phosphorylation, that are crucial in DNA repair, cell cycle regulation, apoptosis and genome stability (120–122). A well-known radiation-induced histone modification is phosphorylation of histone H2AX, often used as a measure of radiation-induced DSBs, which is crucially important for the repair of DNA double strand breaks (DSB) and for the maintenance of genome stability.

The third type of epigenetic radiation-induced modification involves non-coding RNAs (ncRNAs), in particular microRNA (miRNAs). Since their discovery in 1993 (123) miRNAs, which are small RNA molecules, usually 21–23 nucleotides, are emerging as important modulators in many cellular pathways, including gene expression. *In vitro* and *in vivo* studies demonstrate that miRNA expression levels change in response to radiation, and that certain miRNAs alter radiation sensitivity (124–127). Expression levels of a variety of miRNAs after low-LET ionizing radiation are reviewed and listed in Marta et al. (128).

Many findings indicate that DNA methylation, histone modification and miRNA expression are not separate and independent events, but that there is a strong interplay between them. A cross-talk between DNA methylation and histone modification occurs in specific gene loci in a variety of organisms, and DNA methylation can be regulated by miRNAs and vice versa, achieving a sort of mutual regulation [see also the review in (110)]. Also other non-coding RNAs were found to interact with DNA methylation and histones (129).

Interestingly, there is evidence that epigenome may be differently affected by low- and high-LET radiation. Indeed, a number of studies show that exposure to high-LET radiation can result in lasting changes in the total levels of DNA methylation and in the miRNA expression that may be different from those induced by equivalent doses of low-LET radiation (89, 130–135). Some of these studies were focussed on the effect of high energy Fe-ions, as they are representative of the most detrimental component of space radiation for astronauts in deep space. Overall, these experiments show a complex picture, as the observed differences may likely be related not only to radiation quality, but also to differences in biological system, doses/dose rates, time of observation, and assay used [see the reviews in (107, 110)].

In general, irradiation with protons and high-LET heavy ions (Si-, Fe-, Ti-ion) often, though not always, causes hypermethylation in global and/or repetitive element and alterations at specific genes [as reviewed in (135)]. It is interesting to note that, since protons of relatively low-LET gave an effect similar to that caused by high-LET Fe-ions, it appears that epigenetic responses may be related to radiation quality (i.e., to properties in their track structure) rather than to LET (130). Experiments with human primary lung cancers suggested that exposure to these particle “creates a DNA methylation ‘signature’ that uniquely reflects cancer-specific methylation patterns” (135). The difference between sparsely and densely ionizing radiation has been ascribed to the possible difference in oxidative stress (130). It was also speculated that an increased DNA methylation might occur because of the higher persistence of lesions in heterochromatin related to inefficient repair of clustered lesions along the particle trajectory of densely ionizing radiation (135).

An important question concerns the possibility of transgenerational epigenetic effects in organisms exposed to ionizing radiation. In cells, relatively stable epigenetic modifications were observed that are heritable through mitosis, and can manifest in the progeny of the irradiated cells for many divisions (63). In plants and in some animals, such as nematodes, transgenerational epigenetic inheritance is relatively common and many examples are reported (136) but in mammals epigenetic patterns are largely erased and then remodeled during germ cell development and early embryonic development (epigenetic reprogramming) (137, 138). Nevertheless, transgenerational induction of chromosomal instability has been documented in irradiated rodents (63, 139, 140) but the occurrence of transgenerational radiation effects is highly controversial in humans [see the review in (110)].

##  Links Between Radiation-Induced Oxidative Stress, Epigenetic Changes, Non-Targeted Effects and Adaptive Response

Ionizing radiation induces oxidative stress when excess of ROS/RNS are not compensated by the scavenging mechanisms of the cell. ROS can be directly generated by radiation exposure and indirectly through the damage of mitochondria (141).

Besides the well-known mutagenic action of ROS and RNS, there is also evidence that oxidative stress can modify the epigenome by multiple mechanisms, the most important of which involve oxidation of DNA bases and/or mitochondria-mediated changes, with the main target being the CpG sites, especially in the CpG islands (142, 143).

Oxidative stress are also known to play an important role in NTE and AR (144, 145). For example, they contribute to genomic instability (146) and may spread to neighboring, non-targeted bystander cells (27). It appears therefore that they share the radiation-induced oxidative stress as a common origin with the radiation-induced epigenetic changes.

**Figure 4:**
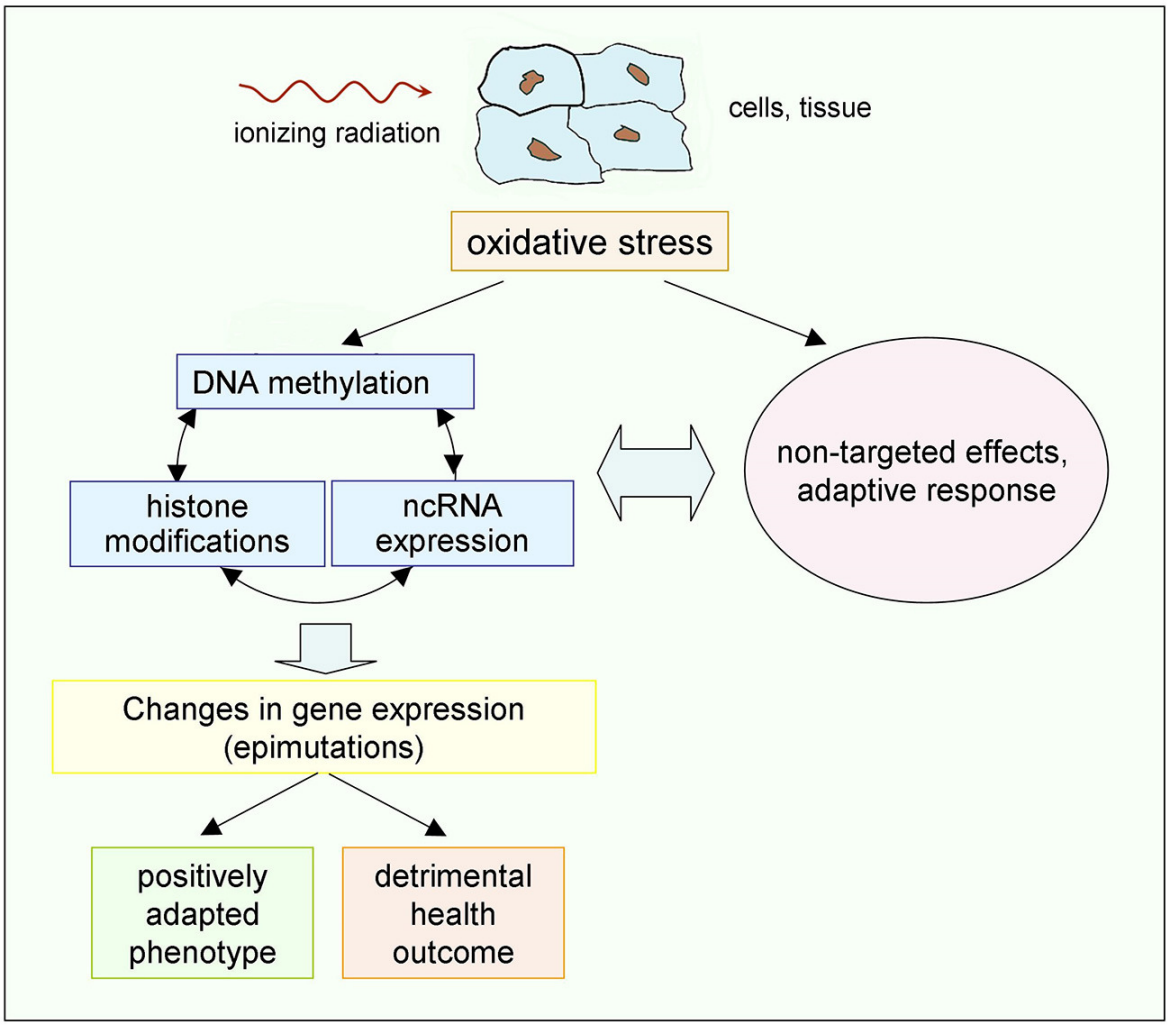
Possible chain of events driven by low doses of ionizing radiation. Epigenetic mechanisms, on the one hand, and non-targeted effects and adaptive response, on the other hand, share as a common origin the radiation-induced oxidative stress, and interplay exists between them (89, 90). The resulting effects may, in principle, either provide a positively adapted phenotype or a detrimental outcome (45). Occurrence of additional radiation-induced genetic effects, not shown here, cannot be excluded, but gene mutations are much less frequent than epimutations [see the review in (147)].

The existence of inter-relations between BE, GI, and AR, from one side, and of those between DNA methylation, histone modifications and ncRNA expression, from the other side, along with the existence of interactions among all these processes, speaks in favor of a picture where the biological response to ionizing radiation is a complex response to a variety of signals at the cellular and supracellular levels. In this picture, epigenetic changes have become increasingly recognized as important aspects besides the genetic ones, especially at low doses (Figure 4). It is then plausible that this complex response explains the observation of non-linear phenomena. These considerations strongly suggest that the assumption of a linear dose-response, at least at low doses, for radiation- induced cancer is an oversimplification. Developing more realistic radiobiological models is not only relevant to radiation biology, but is also important in radiation protection to guide extrapolations to low doses and low dose rates of epidemiological data on exposed human populations, and also to identify the factors determining individual radiation sensitivity/susceptibility. Realistic models, focused on biological end-points relevant to health effects in humans, could also settle the long-standing dispute about possible “beneficial” effects of low doses, such as “radiation hormesis,” i.e., an induction of beneficial health effects by stimulation of defense mechanisms by low-dose radiation.

## Underground Radiobiology Experiments

Understanding the role of natural background radiation on living organisms is essential to complete the above mentioned scenario, given that this background contributes to the basal biological state on which the response to additional (man-made) exposures superimposes. It is recognized that studies of low-dose-rate exposure from environmental sources can potentially contribute to a better understanding of the risks of radiation-induced cancer (7). It is interesting to note that the background radiation shows large geographical variations (3) and that residents in high natural background radiation (HNBR) areas were the subject of several epidemiological studies in the attempt to find possible differences between them and people living in normal background areas. Overall, these studies did not show any increase in cancer risk, but since they are subjected to the already mentioned typical limitations of epidemiological studies (mainly lack of statistical power and biases and confounding factors) it was recognized that improvements would be needed (7). Residents in HNBR areas were also the subject of cytogenetic investigations. For example, in the Ramsar area of northern Iran, one of the areas with the highest radiation background in the world, preliminary findings suggested no significant difference in cytogenetic parameters in the residents there compared to people living in normal background areas (148), but subsequent studies showed a higher incidence of chromosomal aberrations (149) and of spontaneous levels of DNA damage and radiosensitivity in the study group compared to the control groups (150, 151). In spite of the recognized potential contribution of HNBR studies to a better understanding of the risks of radiation-induced cancer (7), the data so far obtained are far from providing a consistent picture and a solid contribution to the matter, so that various hypotheses are still on the table. For example, lack of proven detrimental health effects in the Ramsar residents was hypothesized as due to induction of adaptive response and, consequently, to the induction of non-linear responses (152).

Controlled long-term experiments with model organisms, conducted in underground laboratories where conditions with no or largely reduced radiation background are realized, compared with normal conditions at natural background radiation, can provide basic information for understanding the role of this background [see the review in (153)].

From the radiobiological point of view, these experiments provide an original approach to the low dose issue, since they aim at investigating the effect on biological systems after removing or decreasing the radiation dose, rather than observing the effect after exposing them to small doses that superimpose to the background. The latter case implies an exposure increase over the (local) natural background and, if the dose increments used in these conventional experiments are comparable to the dose coming from the natural radiation background, its geographical variations may make inappropriate any comparison between different laboratories.

Underground laboratories were mainly built to host experiments in fundamental Physics and Astrophysics searching for rare events such as neutrino interactions, thus requiring a very low radioactivity environment to decrease the experimental noise (154). The overburden provides a shield that strongly reduces the cosmic ray flux and in several cases additional measures were undertaken to reduce other components of the radiation background. A list with the relevant characteristics of these underground laboratories around the world can be found in Morciano et al. (155). Only quite recently their unique opportunities for radiobiology experiments were exploited in some of them. For a survey of deep-underground laboratories focusing on biological research see the review in Liu et al. (156). So far, a number of studies have been carried out in several underground laboratories set up in various countries using a variety of cell and organisms, i.e., bacteria, protozoa, yeasts, rodent and human cells, fruit flies (153).

Pioneering works carried out in a low-radiation environmental laboratory in the Pyrenees Mountains, have shown that the growth rate of paramecium cells was decreased when they were cultured in low background radiation. Normal growth was restored upon the addition of a radiation source equivalent to control levels (157, 158).

Two bacterial species, *S. oneidensis* and *D. radiodurans*, grown under reduced radiation environmental conditions at the Waste Isolation Pilot Plant in New Mexico, have shown upregulation of oxidative stress-related genes and of heath-shock protein genes, respectively, and reduction of growth rate for both species, suggesting that a stress response is triggered in the absence of normal levels of radiation. Interestingly, these changes were restored after the recovery of radiation (159). Also, up-regulation of oxidative stress related proteins was observed in human lung fibroblast cells and bronchial epithelial cells grown in low radiation background (160, 161). Transcriptome analysis in *S. oneidensis* deprived of background radiation showed down-regulation of ribosomal proteins, indicating a marked decrease in protein translation, and a genome-wide gene regulation response that was interpreted as due to a lower intracellular radiolysis products concentration because of a reduced hit rate by radiation tracks (162).

In the 1990s a series of experiments were started in the Gran Sasso underground Laboratory of the Italian National Institute of Nuclear Physics, shielded by about 1,400 m of rocks. The first study, carried out on the yeast strain *S. cerevisiae*, showed that permanence in the low radiation laboratory decreased the cell defense ability against genotoxic and radio-mimetic agents (163). Subsequent experiments, using rodent and human cell lines, cultured for 9 months and over, showed that cultures kept in this strongly reduced background, when compared with similar cultures in normal background, showed less ROS scavenging ability, down-regulation in genes involved in protection from oxidative damage, and higher susceptibility to subsequent radiation-induced damage (164–166). As a step forward the study of more complex organisms, the investigators started studying the fruit fly *D. melanogaster*, providing the first evidence of the influence of the radiation environment on life span, fertility and response to genotoxic stress at the organism level (167). Interestingly, permanence in low radiation environment increased the fly life span, but decreased male and female fertility, and trans-generational effects were demonstrated.

Despite the difficulties of ruling out confounder factors when comparing the underground situations to the normal ones (i.e., factors such as temperature, humidity, atmospheric pressure, etc.), on the whole these results suggest that the natural background radiation is capable to stimulate defense mechanisms against stress, including further exposure to ionizing radiation. The observed effects on oxidative stress genes, and the restoration after radiation recovery, are consistent with the hypothesis that epigenetic mechanisms are involved in setting up or reinforcing these defense mechanisms. It is worth noting that in some of the mentioned experiments (165, 166) the possibility was ruled out, through parallel analysis of multiple samples, that the results were affected by random selection of (genetic) mutants, which corroborates a possible involvement of epigenetic mechanisms.

## Conclusions and Perspectives

Awareness that we live in a world in which natural background radiation is present everywhere has raised several fundamental questions, from the general one “does life need low level radiation ?” to the more specific issues “is natural background radiation a damaging or stimulatory agent ?” and “are epigenetic mechanisms important to the biological response to this background ?”

Observation of radiation-induced biological phenomena such as non-linear responses at low doses (in particular NTE and AR), of epigenetic mechanisms capable to respond to changes in environmental ionizing radiation, and of possible stimulation of cell defense mechanisms by natural background radiation speaks in favor of the following two lines of conclusions that are inter-related.

The first one is the general observation that natural background radiation was, and likely continues to be, essential for evolution of life on Earth by, e.g., stabilizing the genome and, at the same time, allowing the necessary adaptation of organisms to environmental changes. Radiation-induced epigenetic modifications are important players in these processes as it is known that, in general, the epimutation rates can be much faster than rates of (genetic) mutations and the epimutations are more easily reversible (168).

The second and more specific one is related to radiation protection issues. Biological response at low doses appears to be more complex then that taken as a basis for the LNT relationship between health risk and dose, whereby ionizing radiation always induces harmful gene mutations in direct proportion to the energy deposited by radiation. Linear dependence of the health risk on doses above natural background must be seen as an oversimplification. Besides the possibility that radiation-induced epigenetic mechanisms contribute to cancer induction, also the possibility must be considered that this same background triggers a hormetic response, as shown in Figure 1.

The latter consideration highlights some practical drawbacks in the current system, derived from the conclusion that, whatever the real dose-response relationship will be, it is unreasonable assuming a linear one. An important example is in the “optimization of protection,” one of the three fundamental principles of radiation protection (18), where different options, in the current system, could be compared based on collective dose, irrespective of the fact that it can be made of many small individual doses or of few larger ones. At the same time the problem is posed of how health risk can be assessed not only for workers and members of the public exposed to low doses above the background, but also for people working in underground sites at reduced natural background radiation, so that a special branch of medicine (“underground medicine”) has been envisaged (156).

The complex biological response to low and protracted doses, such as those involved in exposure to natural background, calls for a revision of the relevant radioprotection approach as currently assumed by the International regulatory bodies. It can be noted that even though non-linear, epigenetic-mediated, responses such as the GI, BE and AR, are already incorporated in epidemiological measures of risk (18, 68), nevertheless these responses, as well as epigenetic mechanisms in general, may be relevant at low doses where the epidemiological data are very uncertain. In this region any realistic radiobiological model used for extrapolating the epidemiological data should include both genetic and epigenetic effects induced by ionizing radiation. Development of such new models requires, besides *in vitro* studies that continue to be important for a better understanding of the mechanisms of radiation action, also *in vivo* investigation on model organisms suitable for plausible extrapolation of the results to the human beings. In this research area “underground biology” appears as a new and exciting area for improving our knowledge in evolutionary biology and fundamental radiobiology, as well as in its practical applications, such as radiation protection. More relevant information is expected to be gained from the ongoing research in the already mentioned laboratories (France, USA, Italy) and in others in Canada, Spain and China [see the review in (156)]. An interesting aspect that could be addressed by these investigations concerns the role of the high-LET component of background radiation, exploiting the possibility of simulating the dose-rate at the surface using weak gamma-sources which, however, lacks the high-LET cosmic component. Moreover, underground experiments could be combined with observations coming from HBRAs to get information on the role of background radiation and its various components by comparing exposures spanning from well below the average background dose-rate to several times it.

It is expected that such developments can settle the long-standing controversy about the detrimental or beneficial effects of low-level exposures. Indeed, this is not a trivial question, as every coin has two sides. Although stimulation of defense reactions is evocative of a positive outcome, it could not be the case if, for example, cells damaged by protracted exposures at low doses escape apoptosis, a situation that could enhance tumor promotion by increasing the probability of survival of cells with accumulating damage or mutation (49). Therefore, settling this controversy needs deeper insights of those radiobiological genetic and epigenetic mechanisms that dominate at low doses and at the same time are relevant to health effects on humans, so as to avoid biased, and even opposite, conclusions based on “cherry picking” of published data. It should also be considered that in human studies the individual radiosensitivity/susceptibility can be a source of large variability around the average responses as those represented in Figure 1.

As a final consideration about the process of improving the current system of radiation protection, we cannot overlook that the latter has the advantage of being manageable since, e.g., a given dose can be used as a direct index of risk and different doses can be summed up to evaluate the overall risk. Keeping an acceptable level of manageability is an important constraint in the process of replacing the current paradigm with a new and more realistic one.

## Author Contributions

MB: conceptualization of the work, writing the original draft, and manuscript supervision. LI: bibliographic resources and revising the manuscript. All authors have read and agreed to the published version of the manuscript.

## Conflict of Interest

The authors declare that the research was conducted in the absence of any commercial or financial relationships that could be construed as a potential conflict of interest.

## Footnotes

^1^LET stands for Linear Energy Transfer, defined as the ratio between the energy Δ*E* deposited by a charged particle in a very short track element, and its length Δ*x*. It also corresponds to the linear collision stopping power of a charged particle in a medium. For radiation other than charged particles (e.g., X- or γ-rays, neutrons) it is often conventionally defined by the average LET of their charged secondaries. More details are found in the Report 85 of the International Commission on Radiation Units and Measurements, ICRU (5). Since the biological effectiveness of radiation is related to ionization density, LET is currently considered as an index of it. However, as LET is a measure of linear ionization density, not of spatial density, the terms “sparsely ionizing/densely ionizing radiation” are sometimes preferred instead of “low-/high-LET radiation.”

^2^The sievert (Sv) is the unit of equivalent dose obtained by the unit of absorbed dose, gray (Gy), by applying (a) the radiation weighing factors to take into account the biological effectiveness of the different radiation qualities, and (b) the tissue weighing factors to take into account the inhomogeneity of the exposure. For the special case of uniform total-body exposure to low-LET radiation: 1 Sv = 1 Gy.

## Abbreviations

UNSCEAR: United Nations Scientific Committee on the Effects of Atomic Radiation
ICRP: International Commission on Radiological Protection
DSB: Double strand break
LET: Linear energy transfer
LNT: Linear No-Threshold
NTE: Non-targeted effects
AR: Adaptive response
BE: Bystander effect
GI: Genomic instability
C, G: Cytosine, Guanine
CpG: 5'—C—phosphate—G−3'
DNMT: DNA methyltransferase
ncRNA: Non-coding RNA
miRNA: Micro RNA
ROS: Reactive oxygen species
RNS: Reactive nitrogen species
TE: Transposable element
HNBR: High natural background radiation.
